# Shear wave propagation in the Achilles subtendons is modulated by helical twist and non-uniform loading

**DOI:** 10.1098/rsos.241058

**Published:** 2025-06-18

**Authors:** Jonathon Blank, Lauren Welte, Jack Martin, Darryl Thelen

**Affiliations:** ^1^ Department of Mechanical Engineering, University of Wisconsin–Madison, Madison, WI, USA; ^2^ Department of Mechanical Engineering, University of Alberta, Edmonton, Alberta, Canada; ^3^ Department of Biomedical Engineering, University of Alberta, Edmonton, Alberta, Canada; ^4^ Department of Orthopedics and Rehabilitation, University of Wisconsin–Madison, Madison, WI, USA; ^5^ Department of Biomedical Engineering, University of Wisconsin–Madison, Madison, WI, USA

**Keywords:** tendon morphology, triceps surae, finite element model, gait simulation, noninvasive stress measurement, Achilles tendon anatomy

## Abstract

The triceps surae is composed of the medial gastrocnemius, lateral gastrocnemius and soleus muscles. Each muscle inserts onto a subtendon that undergoes helical twist prior to insertion onto the calcaneus. Shear wave tensiometry is a non-invasive technique to gauge Achilles tendon loading, yet it is unknown whether subtendon loading can be resolved using subtendon-specific shear wave speeds. The objective of this study was to examine shear wave propagation in the lateral gastrocnemius, medial gastrocnemius and soleus subtendons of the free Achilles tendon. We expected to show that the helical arrangements of subtendons within the Achilles would modulate wave propagation, and that non-uniform loading between subtendons would elicit nonuniform wave propagation. We created a finite element model of the Achilles tendon and simulated shear wave propagation. We found that helical subtendon twist had little effect on wave propagation speed. When a two-fold stress differential was applied to the gastrocnemius and soleus subtendons, the shear wave speed-axial stress relationship was modulated by adjacent subtendon tension and the amount of overall subtendon twist. These findings enhance the basis for tensiometry in the Achilles tendon and inform causes for variability in shear wave speeds measured using shear wave tensiometry or elastography.

## 1. Introduction

The Achilles tendon is a primary load-bearing tendon in human locomotion and is composed of three subtendons that arise from the lateral gastrocnemius, medial gastrocnemius and soleus muscles. The subtendons undergo helical twist prior to insertion onto the calcaneus, with the degree of twist varying substantially between individuals [1,2]. Thus, variation in the biomechanics of the composite structure of the Achilles tendon between individuals may be altered by the morphology of underlying subtendons [3,4]. There is also indirect evidence of differential loading among subtendons, including imaging-based measures of non-uniform deformation within the Achilles tendon [5,6] and both differential activation and stretch of the individual triceps surae muscles [7]. However, it remains challenging to use such measures to infer loading *in vivo*, as muscle mechanics, anatomical factors and load transmission between subtendons [8] can complicate the relationship between triceps surae activation, tendon deformation and loading.

Shear wave tensiometry is an emerging technique for gauging Achilles tendon load based on the propagation speed of shear waves [9,10]. In tensiometry, an external actuator induces a transient wave in a superficial tendon. Skin-mounted accelerometers or high-frame-rate ultrasound can be used to track wave speeds in the tendinous tissue [11], which in turn can be used to estimate the axial load [9]. A recent tensiometry imaging study reported distinct wave patterns across the depth of the Achilles tendon [12], which likely reflects differential subtendon loading. This observation raises the question of how subtendon interactions may affect tensiometer or ultrasound-based wave speed measures.

Computational models provide a framework for understanding how anatomical features and tissue properties affect wave propagation in biological tissues. Tendinous tissue can be modelled as a transversely isotropic material, in which fibres are embedded in a matrix. We previously showed that regional shear wave speed varies with the square root of the local stress in a transversely isotropic tissue [13]. However, the precise relationship between wave speed and stress depends on the presence and amount of surrounding subcutaneous tissue [14]. While prior modelling studies have investigated the effects of geometric variations in twist and subtendon anatomy on tendon mechanics during locomotion [15–21], there is no prior work that considers how helical twist, adjacent subtendon interactions and differential loading can alter shear wave propagation in the Achilles tendon.

The objective of this study was to investigate shear wave propagation within the Achilles subtendons during simulated walking. We created a finite element model of the Achilles tendon composed of the lateral gastrocnemius, medial gastrocnemius and soleus subtendons. The model is capable of simulating dynamic shear wave propagation patterns when subjected to variable triceps surae muscle forces encountered during walking. We expected to show that shear wave propagation speed varies between subtendons when differential loading is present, but that the overall linear relationship between squared wave speed and axial stress would be preserved.

## 2. Material and methods

### 2.1. Finite element model overview

**Figure 1.**
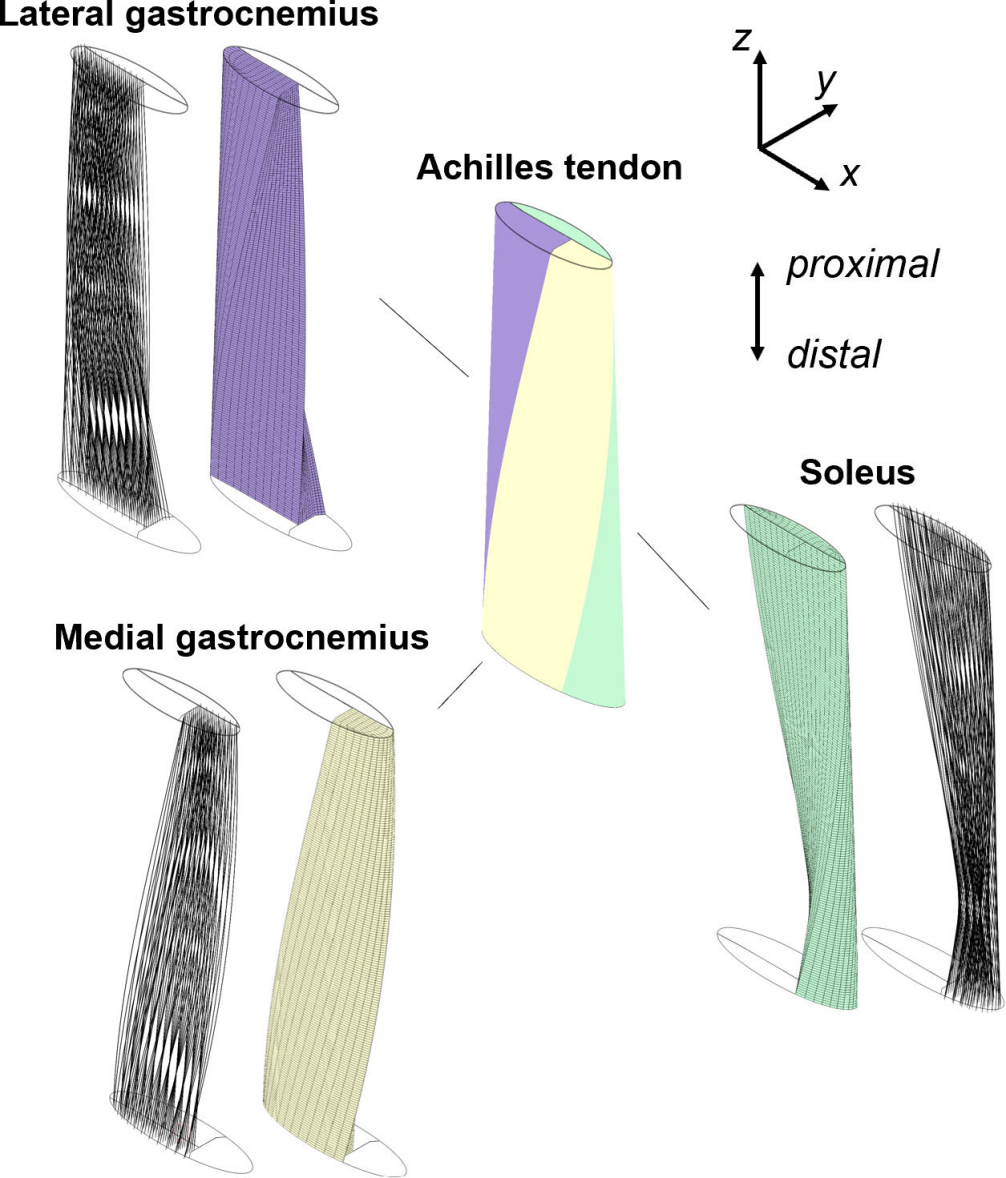
The Achilles tendon finite element model was comprised of the lateral gastrocnemius, medial gastrocnemius and soleus subtendons. Subtendon cross-sectional areas and twists were based upon measurements in a prior study of the left Achilles tendon [3]. Twist was applied to the distal cross-section (*xy*) about the centre longitudinal axis of the composite tissue. Each subtendon was represented using embedded fibres (black, shown) that were aligned with respect to the helical twist axis of each subtendon about the centroid of the tissue cross-section. An Achilles model with a transverse helical twist of 90° is shown.

We created a model of the free Achilles tendon in FEBioStudio v3.0 [22]. The entire cross-section was elliptical and the tissue was modelled as having a taper from the distal (*width* = 22.04 mm, *thickness* = 6.42 mm) to the proximal end (*width* = 17.83 mm, *thickness* = 5.76 mm) (figure 1) [19]. The cross-sectional area of each subtendon at the proximal end was based on prior literature [3]. The entire Achilles tendon was composed of hexahedral elements according to a butterfly mesh, with the lateral gastrocnemius having 22 140 elements, the medial gastrocnemius having 13 500 elements, and the soleus having 16 200 elements. The mesh density was prescribed such that the helical geometry of subtendons was captured while minimizing processing time, with guidance from a prior mesh convergence study [23].

**Figure 2.**
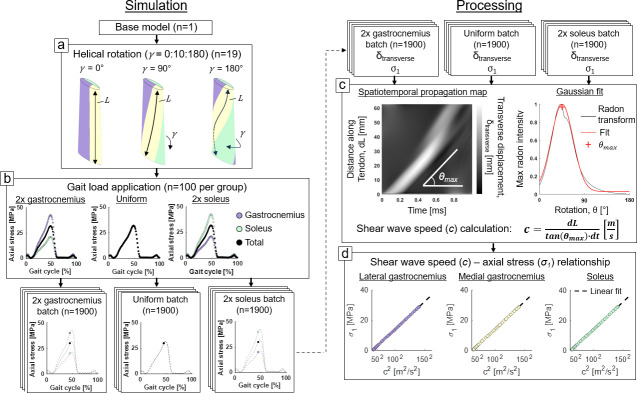
Simulation setup and processing techniques. (a) We generated three batches of 1900 models (19 twist conditions for 100 gait cycle increments) to simulate the effect of helical twist and differential loading on Achilles subtendon shear wave speeds. (b) All three batches of simulations, each batch having a different differential subtendon loading condition, were performed on a high throughput computing grid. (c) We used a Radon transform technique on each subtendon to determine shear wave speeds and (d) assessed shear wave speed-axial stress relationships in each subtendon.

We varied the helical twist of the subtendon bundle using the proximal end as the fixed reference (figure 2a). Helical twist angle was defined as the rotation about the transverse centre axis (i.e. about the *z*-axis) of the entire structure between the proximal and distal end, with the proximal end being held fixed at a 0° reference. We prescribed twist angles between 0° and 180° (in increments of 10°), with variations between 60° and 180° considered to be most anatomically relevant for type I, II and III tendons [3]. Helical twist in the model was generated by rotating mesh nodes about the centre of the composite Achilles tendon cross-section using a custom Python script.

### 2.2. Hyperelastic model

We modelled each subtendon as an incompressible, transversely isotropic and uncoupled hyperelastic material [24] that represents a tissue with longitudinal fibres embedded in an isotropic, Mooney–Rivlin ground matrix [25]. The transverse tangential shear modulus of the ground matrix (and thus the transversely isotropic tissue in the unloaded state) can be estimated as $2\times (C_{1}+C_{2})$, where $C_{1}$ and $C_{2}$ are specified as input (in MPa). The following piecewise function represents the contribution of the collagen fibres to the strain energy density function:


(2.1) $$\begin{array}{c} \tilde{\lambda }\frac{\partial F}{\partial \tilde{\lambda }}=0\;\tilde{\lambda }\leq 1\\ \tilde{\lambda }\frac{\partial F}{\partial \tilde{\lambda }}=C_{3}(e^{C_{4}(\tilde{\lambda }-1)}-1)\;1<\tilde{\lambda }<\lambda^{*}\\ \tilde{\lambda }\frac{\partial F}{\partial \tilde{\lambda }}=C_{5}\tilde{\lambda }+C_{6}\;\tilde{\lambda }\geq \lambda^{*} \end{array}$$

Here, F is the deformation gradient of embedded fibres, $\tilde{\lambda }$ represents the tissue stretch during loading and $\lambda^{*}$ is the stretch at which the fibres engage. Prior to this stretch level, the fibres were either slack or uncrimping, where the term *C_3_* scales the exponential stress and the term *C_4_* controls the strain-dependent rate at which the fibres uncrimp. The term *C_5_* corresponds to the elastic modulus of straightened fibres and *C_6_* is determined using the requirement that the stress-strain curve be continuous at $\lambda^{*}$. Embedded fibre direction was prescribed as parallel with one edge of each rotated element such that fibres were helically wound along the Achilles tendon length. We chose constitutive parameter values based on previous finite element modelling studies of transversely isotropic tendons and ligaments [23,26,27]. The linear region elastic modulus was chosen with guidance from prior *ex vivo* and *in silico* analyses of Achilles tendon mechanics [19,28]. The bulk modulus, K, was chosen based guidance from the finite element software. The tissue density, ρ, was chosen to reflect the wet density of tendon tissue [29]. Each subtendon was modelled using the same constitutive parameters (table 1).

**Table 1.** Finite element model constitutive parameters.

parameter	value
density, ρ [kg m^−3^]	1500
$C_{1}$ [MPa]	2.05
$C_{2}$ [MPa]	2.05
$C_{3}$ [MPa]	1.75
$C_{4}$	50
$C_{5}$ [MPa]	350
$\lambda^{*}$	1.03
K [MPa]	10^3^ $\times C_{1}$

### 2.3. Simulation of achilles tendon loading and shear wave propagation

#### 2.3.1. Static triceps surae loading

We applied static forces to the proximal end of the free tendon with the distal end held fixed. Forces were generated based on overall triceps surae forces during gait that was estimated in a prior study using *in vivo* walking data and a musculoskeletal model [7]. The lateral and medial gastrocnemius were loaded identically, and the soleus was loaded separately. During the entire simulation, contact was maintained between the surface normal of subtendons using a penalty-enforced, frictionless sliding elastic contact. We created three cohorts of models: one with a uniform force applied to the entire Achilles tendon (i.e. no differential loading or relative sliding), and two with a twofold differential axial stress level between the gastrocnemius and soleus subtendons such that the total force in the entire Achilles tendon remained the same (figure 2b). Axial stresses, and thus axial stress differentials, were determined *a priori* using the undeformed cross-sectional area of each subtendon. We estimated individual subtendon forces to achieve the desired axial stress using the cross-sectional area of each subtendon at the distal end. We generated 100 models per cohort, each representing loading at successive one percent increments of the gait cycle. All model files were generated using a custom Python script.

#### 2.3.2. Dynamic shear wave propagation

We excited the shear wave in the Achilles tendon using an impulsive plane wave excitation across the entire distal cross-section, in accordance with a prior study [14]. This idealized excitation was performed by displacing nodes transversely according to a half-sine displacement profile with a half period of 250 μs and an amplitude of 20 μm. The resulting shear wave in the subtendons was simulated for 1 ms at a time step resolution of 10 µs. All shear wave propagation simulations were performed using FEBio v 4.0 [22] on a high-throughput computing grid (Center for High-Throughput Computing, University of Wisconsin–Madison, https://chtc.cs.wisc.edu).

#### 2.3.3. Transient shear wave speed measurement

We measured shear wave speed using the transverse displacement profiles of each node along the cross-sectional centroid of each Achilles subtendon. Displacement patterns were reconstructed into a spatiotemporal map spanning the length of each subtendon and the first millisecond of the dynamic shear wave propagation simulation. We used a directional filter to remove backward-travelling reflections from the proximal end of the structure [30]. Thus, only shear waves that travelled distal-to-proximal were used in the shear wave speed calculation, as is done *in vivo*. We then used a Radon transform to determine the speed of the transient shear wave. The Radon transform can be used to determine the rotation angle aligned with the maximum transverse displacement band in the spatiotemporal wave propagation map (figure 2c). This angle can then be converted to slope, or shear wave speed, in m s^−1^. To approximate the average shear wave speed, we fit a first-order Gaussian function to the maximum Radon transform amplitude at each angle, where the peak of the fitted curve determined the primary propagation angle, which corresponds to the average shear wave speed. We then evaluated the relationship between the measured shear wave speeds and the first principal stress along the centreline of each subtendon (figure 2d).

### 2.4. Statistical analysis

We assessed the shear wave speed-axial stress relationship using the tensioned beam model for shear wave tensiometry [9,31]:


(2.2) $$\sigma =\rho_{eff}c^{2}-k^{\prime }\mu$$

Here, σ represents the axial tendon stress (in Pa), $\rho_{eff}$ the effective density of the propagation medium (in kg m^−3^), c the shear wave speed (in m s^−1^), $k^{\prime }$ a shear correction factor and μ the tangential shear modulus of the tendon (in Pa). We used linear regression to assess the correspondence of model results to our tensioned beam model. For all linear regressions, the dependent variable was the axial stress, *σ*, and the independent variable was the squared shear wave speed, $c^{2}.$

## 3. Results

### 3.1. Non-uniform loading and helical twist distort shear wave propagation patterns in subtendons

**Figure 3.**
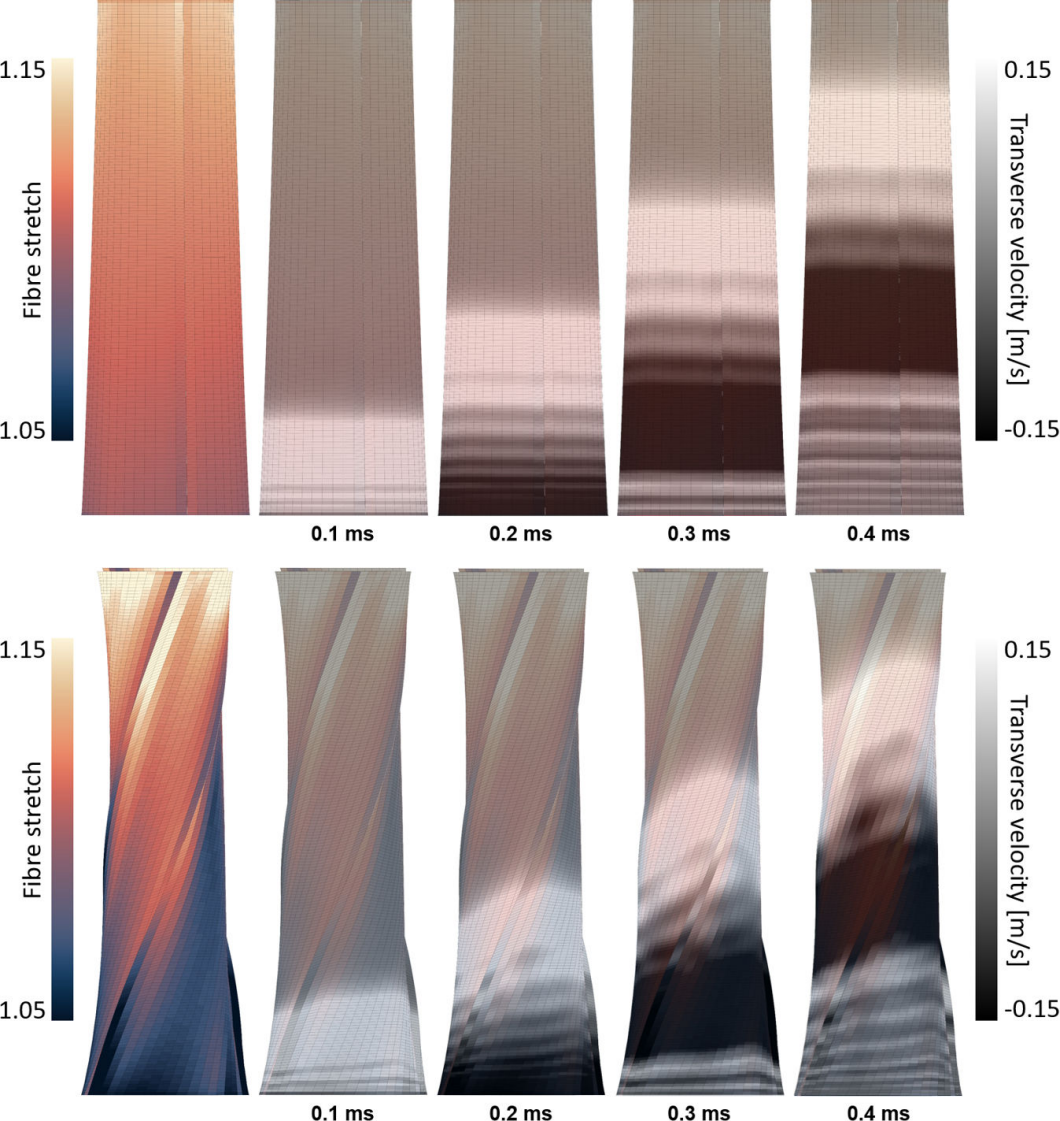
Posterior view of the Achilles tendon fibre stretch and wave propagation (0.1 ms increments) with no helical twist (upper row) and a 180° helical twist (bottom row). With no twist present and uniform loading across tendons, a distally induced wave propagates uniformly along the long axis of the tendon. Helical twist induces nonuniform loading of the subtendons and shear wave propagation patterns. Stress was greater in the proximal region of the Achilles due to the tapering cross-sectional area.

Uniform subtendon loading in an Achilles tendon with no helical twist resulted in uniform shear waves that propagated along the long axis of the tendon (figure 3 top). The introduction of helical twist resulted in nonuniform stretching of the subtendon fibres (figure 3 bottom). Shear wave propagation became less uniform when twist was present.

**Figure 4.**
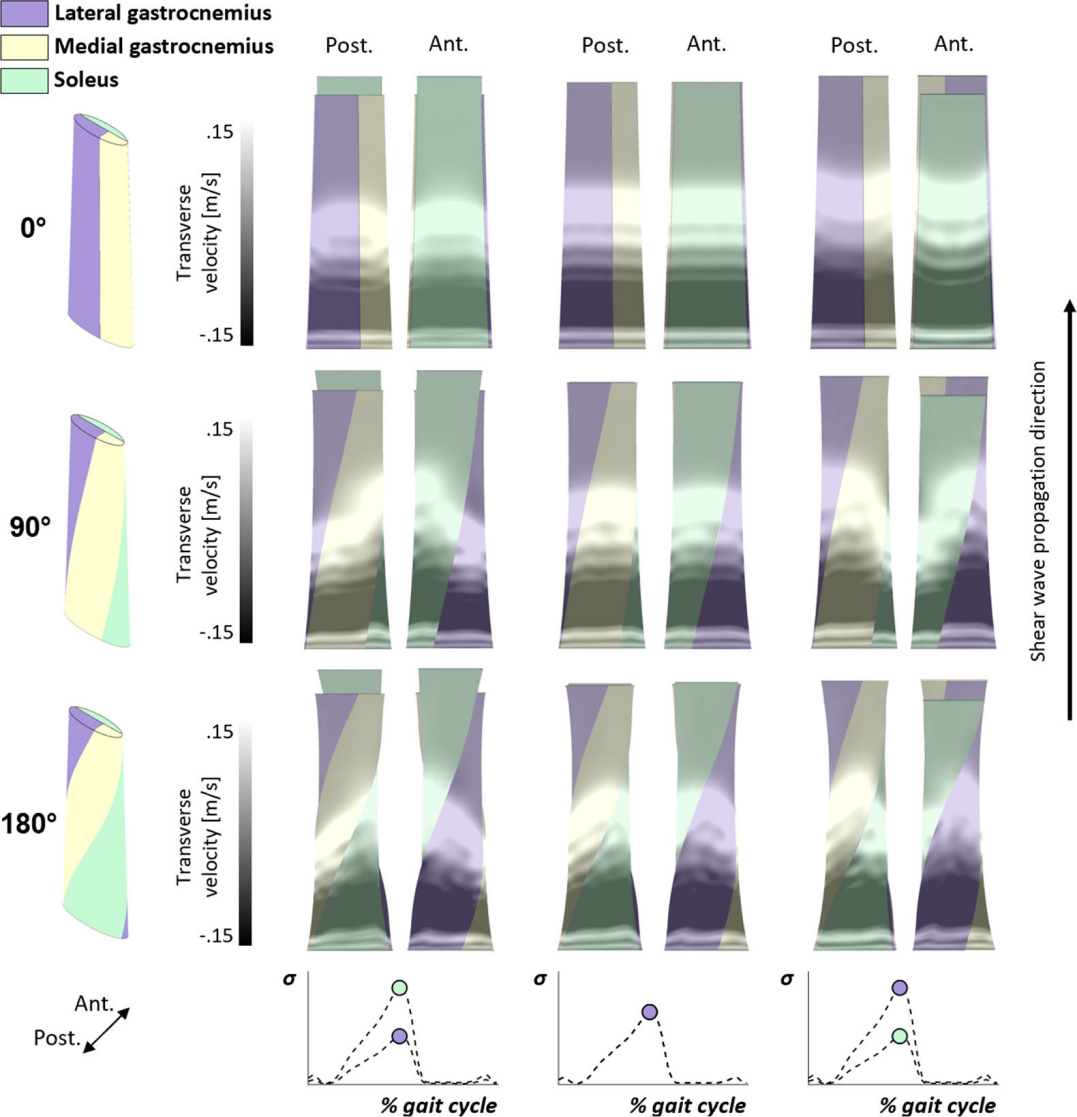
Shear wave propagation colourmaps for Achilles tendons in late stance (high overall loading) at 0.3 ms following the shear wave excitation. Shown are posterior and anterior views. Both helical twist (rows) and non-uniform loading (columns) in adjacent subtendons induced distortions in the wave propagation patterns. For example, greater soleus subtendon loading induced faster propagation in the adjacent gastrocnemius subtendons (upper left), whereas lower soleus loading induced slower wave propagation (upper right). Bottom plots show changes in stress over the gait cycle with dots corresponding to the time point shown in colourmaps.

Shear wave propagation patterns across the tendon suggested that wave propagation speed in a given subtendon is affected by the axial stress state of adjacent subtendons. This is most easily observed in the 0° helical twist models (figure 4 top), where the portion of the shear wave on the lateral and medial edges of the gastrocnemius subtendon (i.e. the portions of the gastrocnemius that are not overlying the soleus) travel slower when the adjacent soleus is preferentially loaded, and faster when the adjacent soleus is less loaded. The combination of helical twist and non-uniform loading further distorted the wave patterns across the tendon, with some variation through the tendon depth on overlapping subtendons (i.e. posterior vs anterior view) (figure 4 bottom).

### 3.2. Helical twist promotes shear wave speed differences between subtendons

**Figure 5.**
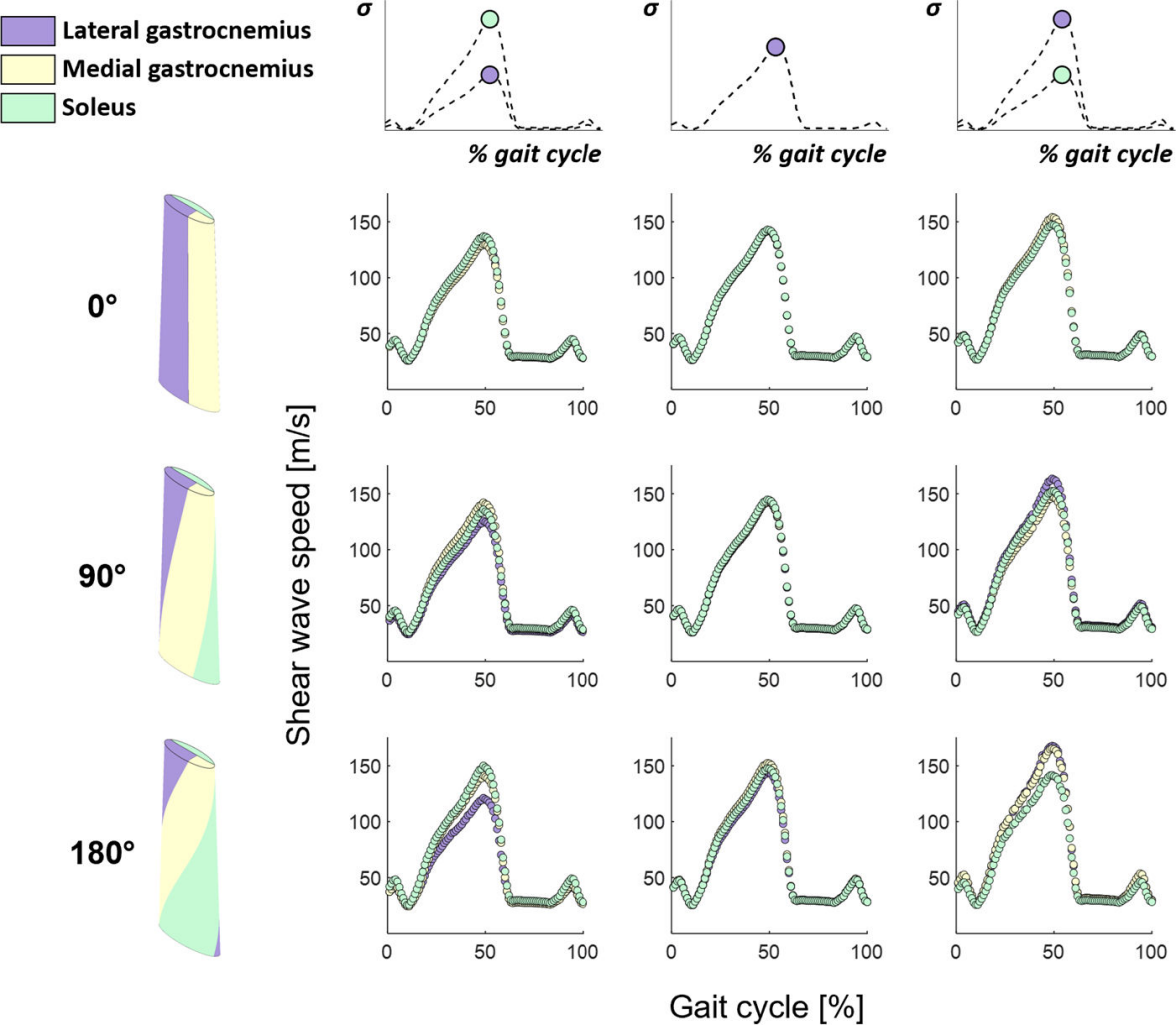
Subtendon shear wave speeds across simulated gait. As helical twist was increased, subtendon shear wave speeds became more disparate during differential subtendon loading, with a maximum 28.9 m s^−1^ difference at peak loads.

Shear wave speeds throughout simulated gait corresponded well with Achilles tendon loading profiles (figure 5). Achilles tendons without twist had similar shear wave speeds across all subtendons for a given load. However, as twist increased, differences in shear wave speeds between subtendons arose, with as high as a 28.9 m s^−1^ difference between subtendons at peak Achilles tendon loading at a twist angle of 180° (figure 5 bottom). Interestingly, the shear wave speed in the medial gastrocnemius subtendon tracked with the more highly loaded subtendon (i.e. lateral gastrocnemius or soleus) even though the applied load was the same as the lateral gastrocnemius. Overall, wave speeds in the adjacent subtendon increased or decreased the wave speed in the subtendon of interest based upon relative load levels.

### 3.3. Differential subtendon loading and helical twist modulate shear wave speed-axial stress relationships

**Figure 6.**
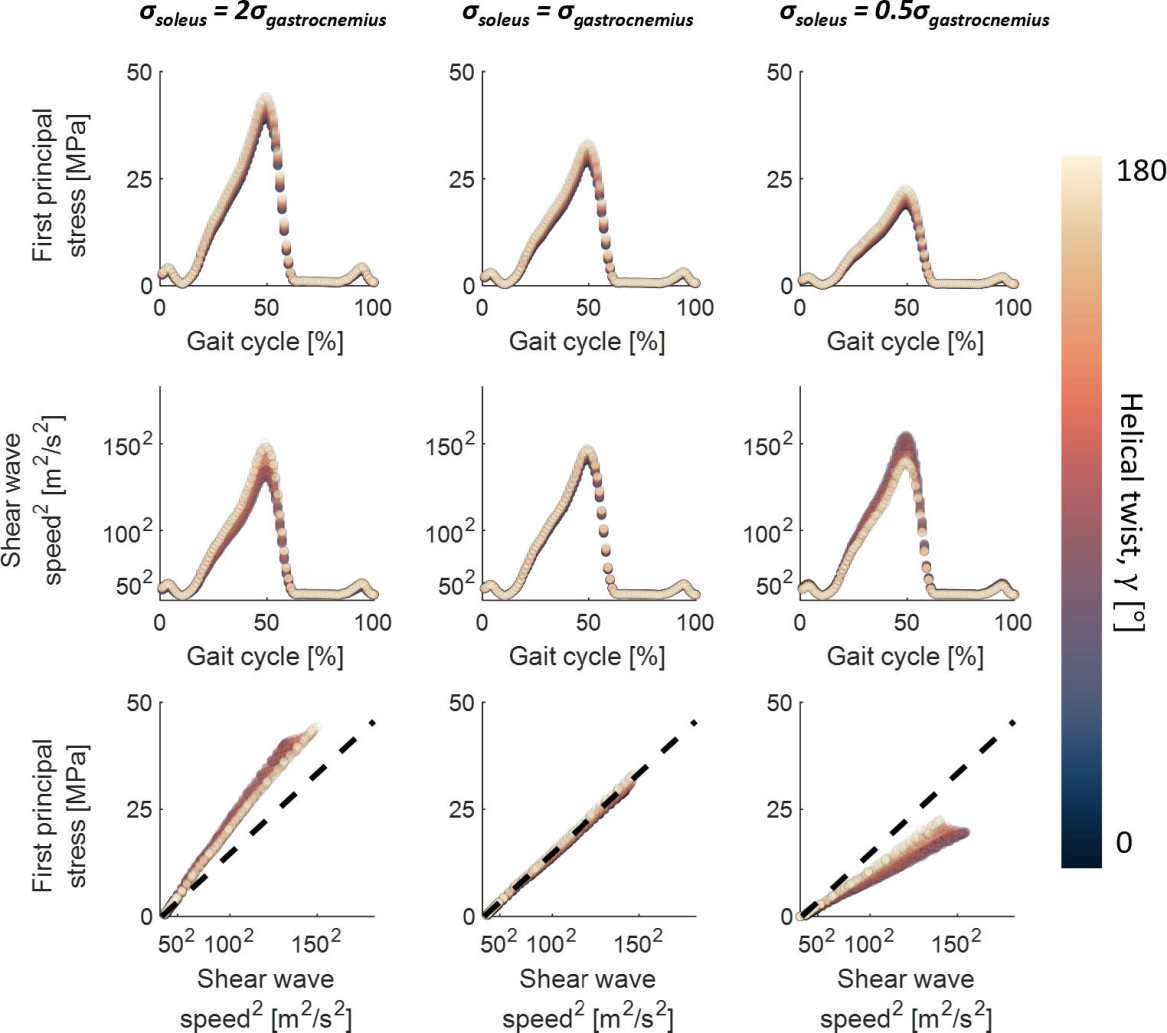
Shear wave speed-axial stress relationships for the soleus subtendon during uniform and non-uniform Achilles loading. Shown are first principal stresses (top) and shear wave speeds (middle) during the simulation, and shear wave speed-axial stress relationships (bottom). The dashed line represents a tensioned beam model with the constant of proportionality assumed to be the density of the tissue. The colour map was chosen to minimize visual distortion [32].

Under uniform subtendon loading conditions, the wave speed-stress relationship was well described by a tensioned beam model (figure 6). There was a strong linear relationship between wave speed squared and axial fibre stress (*R*^2^ > 0.99) that was retained even when helical twist was introduced.

Under differential subtendon loading, helical twist slightly increased the principal stretch and stress along the fibres (figure 6 top). The linear relationship between shear wave speed squared and axial stress was retained (*R*^2^ > 0.99 for all helical twist conditions) (figure 6 bottom), though the proportionality constant differed. More specifically, we measured higher shear wave speeds than predicted by the tensioned beam model when the adjacent subtendon had a higher axial stress, and a lower shear wave speed than predicted by the tensioned beam model when the adjacent subtendon had a lower axial stress. Increased helical twist tended to mitigate this effect, reducing the discrepancy in wave speed-stress relationships between adjacent subtendons. However, the constant of proportionality generally did not correspond to the effective subtendon density (ρ = 1500 kg m^−3^) when differential loading was present (table 2).

**Table 2.** Shear wave speed squared-principal stress slopes (i.e. estimates of effective density) of the gastrocnemius and soleus for different differential loading and helical twist conditions. Bolded text indicates greater than ±10% deviation from the expected slope (1500 kg m^−3^).

helical rotation	σ_soleus_ = 2σ_gastrocnemius_			σ_soleus_ = σ_gastrocnemius_			σ_soleus_ = 0.5σ_gastrocnemius_		
	LG	MG	S	LG	MG	S	LG	MG	S
0°	**1123**	**1160**	**2145**	1465	1465	1454	**1741**	**1703**	**897**
30°	**1173**	**1084**	**2192**	1468	1476	1449	**1666**	**1831**	**873**
60°	**1232**	**1009**	**2241**	1477	1480	1427	1614	**1928**	**817**
90°	**1279**	**996**	**2350**	1495	1482	1472	1570	**1925**	**857**
120°	**1349**	**1052**	**2164**	1522	1468	1480	1546	**1796**	**992**
150°	1418	**1097**	**2051**	1541	1438	1518	1533	**1688**	**1079**
180°	1520	**1116**	**2060**	1583	1417	1551	1563	1625	**1134**

## 4. Discussion

The objective of this study was to investigate the effects of helical twist and non-uniform loading on shear wave propagation within the subtendons of the Achilles tendon. To accomplish this, we created a finite element model of the Achilles tendon that included subtendons of the medial gastrocnemius, lateral gastrocnemius and soleus. We simulated shear wave propagation within the subtendons when subjected to loads that arise during human walking. Subtendon shear wave speeds consistently increased in proportion to the square root of subtendon stress, as predicted by a tensioned beam model. However, when the Achilles tendon was subjected to differential loading across subtendons, absolute wave speeds were modulated by the stress in adjacent subtendons. These insights can be used to inform shear wave tensiometry, which measures wave speeds as a means of noninvasively assessing tendon loading.

The Achilles subtendons undergo helical twist between the proximal triceps surae muscles and their distal insertion onto the calcaneus. Prior finite element modelling studies have investigated the influence of such twists on internal tissue mechanics, concluding that twists can alter the interpretation of two-dimensional mechanics-based measurements [19] and cause nonuniform strain patterns to arise across the tissue and across subtendons [20]. This study used a similar finite element modelling framework to understand how twist affects both internal loading and shear wave propagation within the subtendons. We found that twist induced non-uniform stretch in the fibres, which in turn distorted shear wave propagation patterns. Our simulations also suggest that shear wave speed measurements on an Achilles tendon with less twist reflect an average whole tendon measure, while measurements on an Achilles tendon with more twist reflect individual subtendon loading. It is often assumed that the proximal-distal axis of the Achilles tendon is the primary direction of anisotropy in elastography [33] and tensiometry [9], and it is assumed that there is uniform wave travel during uniform loading. The present evidence of wave distortion and differential subtendon wave speeds as twist is introduced to the model may indicate that more analyses are warranted *in vivo* to determine the sensitivity of transient shear wave speed measurements to transducer or sensor placement along the tissue length and transducer rotation relative to the tissue long axis.

Our primary finding was that differential loading across the triceps surae can modulate the shear wave speed-stress relationship in individual subtendons. However, the fundamental linear relationship between squared wave speed and subtendon axial stress was preserved across all loading scenarios. Under uniform loading, the slope of the shear wave speed-axial stress relationship corresponded closely with the tissue density (*ρ* = 1500 kg m^−3^), which agrees with our prior *in silico* and *ex vivo* tendon studies [23,34]. When the subtendons were differentially loaded, we observed changes in the slope of the shear wave speed-stress relationship within a subtendon. This modulation is dependent on the amount of helical twist present, with greater twist causing more disparate wave speeds but equalizing shear wave speed-stress relationships across adjacent subtendons. For example, in higher twist conditions, the slope of the shear wave speed-axial stress relationship was closer to that of the tensioned Timoshenko beam model (figure 6 bottom and table 2) during non-uniform deformations imposed by differential muscle loading. Further, the constant of proportionality of the squared shear wave speed-axial stress relationship at high helical twist was less disparate between differentially loaded subtendons. This could arise from a more uniform stress within a twisted subtendon during differential loading, an observation consistent with prior studies reporting the equalization of regional loading with increased tendon twist [19,35]. However, the focus of our study was on the sensitivity of shear wave speeds on average to twist and differential Achilles tendon loading. Further study of regional wave speeds within a subtendon may elucidate the interaction of wave propagation with overlying subtendons in the Achilles.

There is evidence of non-uniform Achilles subtendon loading during human locomotion, including differential activation of the triceps surae [7] and non-uniform deformation of the Achilles tendon [6]. Given that tensiometry assesses wave speed as a surrogate measure of tissue loading, it is important to know how wave speed may change with nonuniform loading. Our finding was that shear wave speeds in a given subtendon were decreased when the adjacent subtendon had lower principal stress, and similarly increased when the adjacent subtendon had a higher principal stress. Hence, daily tasks [15,17,36] or joint postures [37,38] that result in differential Achilles subtendon loading may alter wave speeds measured at the sensor location. However, it should be noted that while differential loading affected wave speeds, the change in wave speed due to a twofold difference in axial subtendon stress was modest (figure 5). Further, we assumed either a non-sliding or zero-friction Achilles tendon. The intrafascicular matrix can resist sliding between adjacent subtendons [8] and probably reduce (but not eliminate) the capacity for non-uniform stress to arise between subtendons. This in combination with helical twist may result in Achilles stress that is more uniform across the composite tissue, and hence tendon wave speed measures that have a higher correspondence to *in vivo* axial stress. While we demonstrate the effects of helical twist and differential loading on subtendon shear wave speeds in the present study, additional tensiometry studies are required to determine whether shear wave speeds measured using a wearable sensor superficial to the tendon correspond to superficial subtendon or whole tendon axial stress.

The Achilles and its subtendons exhibit a complex anatomy to help fulfill physiological loading functions. For instance, the cross-sectional geometry of the free Achilles tendon varies in shape along its length in addition to a nonlinear taper [2]. This anatomy also varies between individuals, specifically with regard to cross-sectional area (which can change across age and activity levels) and subtendon twist [3]. In this study, we assumed that the free Achilles tendon had an elliptical cross-section and subtendons that twisted uniformly along the tissue length. Incorporating the aforementioned anatomical features into the model may modify both the axial stress [39] and the resulting shear wave propagation patterns. Thus, while the findings of this study are generalizable towards most applications of shear wave propagation in healthy Achilles tendons, the free Achilles tendon geometry must be appropriately tailored to anatomy when using such a model for personalized or clinical applications.

There are a few limitations to consider when interpreting the results of this study. First, we studied the effect of subtendon loading on wave propagation assuming specific material properties and a generic geometric model of the Achilles tendon. We used the accepted approach of modelling tendons as a transversely isotropic material, which does not account for unaligned fibres and fibril torsion within subtendons [3,19]. However, a prior study found that stress patterns may be more sensitive to geometry than material properties [40]. Our prior modelling studies suggest that unaligned fibres primarily affect wave speeds at low loads [23], which are less relevant to human locomotion. Notwithstanding, a model that accounts for variations in fibre recruitment patterns between subtendons and throughout different subtendon regions, which can be accomplished using flow guides and fluid simulation-based tractography [41], could enhance the physical realism. Second, this study did not explore variations in shear wave speed due to dispersive effects that can arise from viscoelasticity and wave-guided behaviour, where shear wavelengths greater than the finite tendon thickness can contribute to dispersion via guided wave propagation [42,43]. Our prior *ex vivo* studies have shown good agreement between tendon wave propagation and predictions made via an elastic model [23,34], which lends credibility to ignoring viscosity effects in loaded tendons. In this study, we assumed a broadband impulsive excitation and then characterized the resulting transient wave speed of the tissue. Prior analyses suggest that these transient wave speeds correspond with high-frequency phase velocity [14]. Thus, we are confident that shear wave speeds in this study are more readily applicable to our *in vivo* measurement system [9].

This study demonstrates the potential to use spatial variations in tendon wave speeds to infer load distribution within the tissue. An exciting extension of this work would be to use ultra-high framerate ultrasonic imaging to map *in vivo* wave speeds in the Achilles tendon. Using this technology, one could track regional Achilles tendon loading during dynamic movements, which historically has been challenging due to the low framerate of conventional ultrasound elastography systems [44]. A recent implementation of this high framerate technology showed that ultrasound-measured shear wave speeds (40–100 m s^−1^) matched those using shear wave tensiometry *in vivo* (20–80 m s^−1^) [45], and that there are two distinct wave speed patterns in the Achilles tendon during walking, reflecting variations in subtendon loading [12]. There is also the potential for anatomy-based models of shear wave propagation. Prior modelling studies have considered the presence of surrounding subcutaneous fat [14], nonuniform loading within a structure [13] and changes in wave propagation due to changes in tissue microstructure [23]. If combined with an anatomy-informed model, we may move toward providing subject-specific predictions of shear wave speeds under physiological loading that can inform the wide variability in shear wave speed measures across individuals [10]*.* Finally, variation in tensiometer- and ultrasound-measured shear wave speeds *in vivo* can be attributed to user variability, including sensor placement, sensor design (i.e. sensor footprint, ultrasound resolution), transducer pressure and more. Importantly, the current model could be used for simulation-driven sensor design [46] to mitigate variability in sensor-measured shear wave speeds *in vivo* and explore the inherent variability in shear wave speeds of a given sensor across a range of Achilles tendon anatomies.

In summary, we developed a finite element model to investigate physiological mechanisms that alter the shear wave speed-stress relationship in the Achilles tendon measured using shear wave tensiometry. All subtendons had remarkably linear shear wave speed-axial stress relationships independent of non-uniform loading and twist (*R*^2 ^> 0.99). Specifically, we found th following.

— Under uniform loading:
  – Shear wave speeds are similar across subtendons and are independent of twists.
  – The shear wave speed-axial stress relationship holds for all subtendons and is independent of twist.
— Under non-uniform loading:
  – Shear wave speeds are different across subtendons and differences are larger for higher twist Achilles tendons.
  – The shear wave speed–axial stress relationship holds for higher twist Achilles tendons.

Overall, these findings inform the interpretation of Achilles tendon wave speeds measured via shear wave tensiometry or elastography under functional loading conditions.

## Data Availability

Data, simulation files and processing codes for this research work have been archived within the Zenodo repository [47]. Supplementary material is available online [48].
